# 外泌体PD-L1在非小细胞肺癌免疫治疗的研究进展

**DOI:** 10.3779/j.issn.1009-3419.2022.102.33

**Published:** 2022-09-20

**Authors:** 娜 王, 霞 宋

**Affiliations:** 1 030000 太原，山西医科大学第二临床医学院 The Second Clinical Medical College of Shanxi Medical University, Taiyuan 030000, China; 2 030000 太原，山西省肿瘤医院呼吸二科 The Second Department of Respiratory, Shanxi Provincial Cancer Hospital, Taiyuan 030000, China

**Keywords:** 外泌体, PD-L1, 肺肿瘤, 免疫治疗, Exosome, PD-L1, Lung neoplasms, Immunotherapy

## Abstract

肿瘤免疫疗法在当前癌症治疗领域中愈来愈受瞩目，相关研究也层出不穷。对于非小细胞肺癌（non-small cell lung cancer, NSCLC）患者来说，近些年来，以程序性死亡受体1（programmed cell death 1, PD-1）/程序性死亡配体1（programmed cell death ligand 1, PD-L1）免疫抑制剂为代表的免疫检查点抑制剂（immune checkpoint inhibitors, ICIs）已经成为治疗恶性肿瘤的一种最具前景的治疗方案。免疫检查点阻断治疗包括抗细胞毒T淋巴细胞相关抗原4（cytotoxic T lymphocyte-associated antigen 4, CTLA-4）单抗、抗PD-1单抗和抗PD-L1单抗，其中最被人熟知的为PD-L1免疫疗法。目前ICIs在临床治疗中取得了很不错的治疗效果，但有效率较低，因此我们希望获得更高的治疗有效率。近几年外泌体PD-L1在NSCLC免疫治疗中发挥了重要作用。本文就肿瘤外泌体PD-L1蛋白对肿瘤微环境的影响、预测免疫治疗效果以及作为NSCLC免疫治疗的新型治疗策略作一综述。

## Abstract (en)

Cancer immunotherapy is increasingly popular in the field of cancer treatment, and related research is emerging. For patients with non-small cell lung cancer (NSCLC), in recent years, immune checkpoint inhibitors (ICIs) represented by programmed cell death 1 (PD-1)/programmed cell death ligand 1 (PD-L1) immunosuppressants, have become one of the most promising treatments for malignant tumors. Immune checkpoint blockade therapy includes anti-cytotoxic T lymphocyte-associated antigen 4 (CTLA-4) mAb, anti-PD-1 mAb and anti-PD-L1 mAb, with the best-known number of PD-L1 immunotherapy. At present, ICIs have achieved very good therapeutic results in clinical treatment, but with less effective efficiency, so we hope to obtain higher therapeutic efficiency. In recent years, exosomal PD-L1 has played an important role in the progress of immunotherapy for NSCLC. This paper reviews the effects of tumor exosomal PD-L1 protein on the tumor microenvironment, the effect prediction of immunotherapy, and as novel therapeutic strategies for immunotherapy in NSCLC.

对于非小细胞肺癌（non-small cell lung cancer, NSCLC）患者来说，近些年来，除了手术、放化疗传统疗法以外，以程序性死亡受体1（programmed cell death 1, PD-1）/程序性死亡配体1（programmed cell death ligand 1, PD-L1）免疫抑制剂为代表的免疫检查点抑制剂（immune checkpoint inhibitors, ICIs）已经成为治疗恶性肿瘤的一种最具前景的治疗方案^[1, 2]^。尤其对于大多数NSCLC患者来说，在确诊时已有局部晚期疾病或远处转移，晚期患者的5年生存率不足20%，而免疫抑制剂的兴起给NSCLC免疫疗法的实现带来新的临床获益，同时也带来了严峻的挑战^[2-4]^。作为一种新型的恶性肿瘤治疗方法，在一定程度上弥补了传统治疗方式的不足，是充满前景的恶性肿瘤治疗手段。然而，只有一小部分患者获得了长期的临床益处^[5]^。研究^[1]^表明，外泌体PD-L1（exosomal PD-L1, exoPD-L1）正在成为一种非侵入性和容易获得的生物标志物，相比较组织细胞膜PD-L1来说，有着明显的优势。研究^[4]^发现exoPD-L1在免疫抑制和肿瘤进展中起着关键作用，可用于癌症检测的新生物标志物和肺癌的分子治疗靶点以及患者的个体化治疗，对肺癌的免疫治疗具有重要意义。本文综述了近几年国内外有关exoPD-L1对NSCLC免疫治疗反应的研究进展，并进行了小结。

## 1 外泌体的发生

不论是原核生物还是真核生物，几乎所有细胞都会释放细胞外囊泡（extracellular vesicles, EVs）。根据其生物发生、细胞来源和生物学特性可分为三个亚类：微囊泡、凋亡体和外泌体^[6]^。外泌体是起源于核内体，由组织细胞分泌的直径40 nm-160 nm脂质双膜层结构的细胞外囊泡^[6, 7]^。1981年，Trams^[8]^发现了外泌体，他在绵羊网织红细胞体外培养上清液中发现了被双层膜包围的小囊泡。1987年，约翰斯通将这些细胞外小泡命名为Exosomes^[9]^。它始于细胞膜的内吞作用，内吞后形成早期内体（early-sorting endosome, ESE），成熟后形成晚期分选内体（late-sorting endosomes, ISE）。随着内体的成熟，囊泡向内萌发，并在腔内释放，在ISE中形成含有腔内囊泡的细胞内多囊泡体，这些晚期内小体也称为多囊泡小体（multivesicular bodies, MVBs）^[10, 11]^。大多数MVBs与溶酶体融合，进行内容物的降解和循环利用，少数MVBs的膜含有CD63、溶酶体膜蛋白LAMP1、LAMP2，可以与质膜融合，通过胞吐作用释放到胞外，称为外泌体。多泡体形成于质膜的连续内陷，因此可以与其他细胞内囊泡和细胞器相交，从而导致外泌体成分的多样性，包括DNA、RNA、脂质、代谢物以及细胞溶质和细胞表面蛋白^[6, 11-13]^。在肿瘤学领域，研究已经确定了膜PD-1/PD-L1作为多种癌症类型的免疫治疗有效性和肿瘤耐药的生物标志物的潜在价值^[13]^。近年来，随着蛋白质组学技术的普及，外泌体中蛋白质的组成和功能已被揭示，其在预测肿瘤发生、诊断和发展中的作用受到广泛关注。有证据^[14]^表明，肿瘤细胞可以分泌表面携带PD-L1的外泌体，通过其载体（包括蛋白质、脂质和核酸）调节肿瘤微环境并促进癌症进展，是近几年肿瘤免疫治疗的新热点。

## 2 PD-L1

PD-L1又称分化簇274CD274或B7同源体B7-h1，是由*CD274*基因编码的一种Ⅰ型跨膜蛋白^[15]^。它属于B7配体家族，蛋白质结构也类似于该家族的其他成员，由约290个氨基酸组成，具有两个免疫球蛋白样胞外域，包括免疫球蛋白V样和C样胞外域，具有四个特定的N-糖基化、三个磷酸化位点、一个胞内结构域上的棕榈酰化位点C272和一个泛素化位点K178^[15]^。1992年，东京大学的Tasuku Honjo和他的同事们^[16]^发现了PD-1是T细胞上表达的一种与细胞凋亡有关的膜蛋白，并提出PD-1产物可能有其自身的配体。其他几项后续研究试图揭示PD-1的分子相互作用模式，但直到1999年，一种类似的B7同系物，现在被称为PD-L1，在体外被观察到是人类T细胞反应的抑制物^[17]^。后来，有研究^[15, 18]^发现PD-L1不仅在T细胞上，还广泛表达在多种细胞上，主要在肿瘤细胞、巨噬细胞、单核细胞、自然杀伤（natural killer, NK）细胞、树突状细胞（dendritic cell, DC），也在大脑、角膜和视网膜等免疫特需部位表达。在正常生理条件下，PD-1/PD-L1信号通路的激活与维持免疫耐受、促进T细胞免疫稳态、防止免疫介导的组织损伤密切相关^[19]^。在疾病状态下，PD-L1与其受体PD-1相互作用，传递负信号，抑制T细胞介导的一系列细胞免疫反应，包括启动、生长、增殖和凋亡以及功能成熟等过程^[20, 21]^。研究^[22]^发现，在人类血液中，PD-L1存在三种主要形式，一种形式为表达在质膜PD-L1上，另一种为表达在分泌的细胞外泌体表面，或者作为循环的可溶性PD-L1（soluble PD-L1, sPD-L1）。

从生理上讲，PD-1/PD-L1途径的出现是因为需要控制表达抗原部位的炎症程度，以保护正常组织免受损伤。几乎所有活化的T细胞表面均显著表达PD-1蛋白^[23]^。当T细胞识别靶细胞上主要组织相容性复合体（major histocompatibility complex, MHC）抗原时，就会产生炎性细胞因子来启动炎症这一过程。某些细胞因子导致组织细胞表达PD-L1，抑制T细胞的激活从而导致免疫耐受，即使在可起作用的抗原存在的情况下，仍使免疫系统失去对启动炎症反应的控制^[24]^。在某些肿瘤中，最明显的是在黑色素瘤中，这种保护机制通过PD-L1的过度表达而被破坏；结果发现，它绕过了对肿瘤的免疫反应的产生。PD-1/PD-L1抑制剂在药理上阻止PD-1/PD-L1的相互作用，从而促进积极的免疫反应以杀死肿瘤。在肿瘤细胞和免疫细胞中PD-L1分子均有表达。据报道，与肾细胞癌和黑色素瘤相比，NSCLC中PD-L1的表达水平显著升高^[22]^。一般来说，肿瘤细胞上显著的PD-L1表达和高水平的活化T细胞与较高的应答率相关。肿瘤细胞中PD-L1的表达与患者对ICIs的反应有良好的相关性。此前的研究^[25, 26]^证实，PD-L1的高表达水平与PD-1/PD-L1抑制剂治疗后的无进展生存期（progression-free survival, PFS）和总生存期（overall survival, OS）呈正相关。

肿瘤细胞PD-L1在临床上被认为是免疫治疗反应的预测因子。PD-L1也是作为目前指南推荐等级量最高的肺癌免疫相关的生物标志物，是临床应用最广泛的预测性标志物。尽管肿瘤组织PD-L1是美国食品药品监督管理局（Food and Drug Administration, FDA）授权的指示剂，但仅凭肿瘤细胞表面PD-L1的表达模式不足以准确预测肿瘤对抗PD-1/PD-L1治疗的反应。使用膜PD-L1存在诸如创伤性活组织检查、肿瘤内PD-L1表达的异质性、无法进行动态观察以及灵敏度有限等缺陷，它有其局限性，因此PD-1/PD-L1表达并非是抗PD-1/PD-L1治疗疗效的最佳预测标志物^[27]^。研究^[27, 28]^表明，循环中的exoPD-L1正在成为一种非侵入性和容易获得的生物标志物，并且是比组织和血浆中的sPD-L1更容易检测和更可靠的代替性指标。

## 3 exoPD-L1在肿瘤微环境中的作用

在肿瘤微环境中，PD-1及其配体PD-L1通过诱导细胞凋亡和介导免疫逃逸在肿瘤进展和生存中发挥重要作用^[28]^。通常认为PD-L1在瘤床内与肿瘤浸润性淋巴细胞（tumor-infiltrating lymphocyte, TIL）表面的PD-1相互作用。然而，PD-L1也存在于EVs表面，作为膜结合型PD-L1的替代品，肿瘤细胞产生的exoPD-L1在抗肿瘤免疫反应中也发挥着重要的调节作用，并且其PD-L1水平与肿瘤进展有关^[27]^。

Peinado等^[28]^研究发现B16-F10外泌体对骨髓（bone marrow, BM）细胞的作用使Lewis肺癌的原发肿瘤生长和转移负荷增加了10倍，强调了肿瘤外泌体对BM细胞在调节肿瘤发展和转移中的关键作用。有研究^[29]^发现从肿瘤细胞上清液和癌症患者血浆中获得的外泌体部分中分离出肿瘤来源的外泌体（tumor-derived exosomes, TEX），然后利用Western blot证实了各种免疫抑制分子的表达，包括死亡受体配体如FasL或TRAIL、检查点受体配体如PD-L1、抑制性细胞因子如白介素10（interleukin-10, IL-10）和转化生长因子β1（transforming growth factor β1, TGFβ1）以及前列腺素E2和参与腺苷途径的胞外酶CD39和CD73等。癌症患者中几乎所有的循环T淋巴细胞都表达PD-1^[30]^。因此，它们通过携带FasL或PD-L1的膜形式的TEX导致细胞凋亡，并且发现这些凋亡诱导分子在TEX中的表达水平与凋亡频率相关。其次，肿瘤微环境中游离形式的PD-L1除了exoPD-L1还有sPD-L1，而exoPD-L1与sPD-L1相比，其形式更稳定，不易被蛋白水解酶水解，可更强烈地诱导T细胞凋亡。

外泌体可以进入血液和淋巴系统并在全身传播，可以潜在地影响远处部位的肿瘤生长。阻止exoPD-L1的释放不仅会抑制局部肿瘤细胞的生长，还会同时或几个月后阻止远处肿瘤细胞。因此，激活局部淋巴结处的T细胞可产生持久的全身性免疫反应，不再受胞外体PD-L1分泌的影响，最终结果是延长了小鼠的存活期^[31, 32]^。Yang等^[31]^探讨了胞外体PD-L1是否具有与细胞表面PD-L1相似的抑制T细胞活性的功能。在Raji B细胞将抗原呈递给Jurkat T细胞的情况下，体外通过白介素2（interleukin-2, IL-2）的分泌测量PD-L1功能来反映T细胞的激活，结果是在Raji B细胞中没有外源性表达PD-L1的情况下，IL-2分泌量很高。然而，PC3细胞外PD-L1的引入重新抑制了IL-2的分泌，表明来自PC3细胞的exoPD-L1可以替代Raji B细胞上PD-L1的外源性表达^[32]^，可见exoPD-L1具有可以替代细胞PD-L1抑制T细胞激活的功能^[31]^。

Yang等^[31]^通过连续离心法从人乳腺癌细胞MDA-MB-231231和表达PD-L1或*PD-L1*基因敲除PD-L1^KO^的4T1小鼠乳腺肿瘤细胞培养上清液中分离出外泌体。为了评估exoPD-L1在肿瘤微环境和体内肿瘤进展中的作用，测量了联合注射exo-PD-L1^Flag^、exo-PD-L1^KO^或PBS的4T1-PD-L1^KO^细胞的肿瘤生长，结果表明4T1-PD-L1^KO^细胞中缺乏PD-L1导致肿瘤实质性退化；然而，exo-PD-L1^Flag^显著恢复了4T1-PD-L1^KO^细胞的肿瘤生长。为了进一步探索exoPD-L1是否抑制CD3/CD28触发的T细胞激活信号通路，研究者用植物血凝素（phytohaem agglutinin, PHA）处理外周血单个核细胞（peripheral blood mononuclear cell, PBMC）来产生T细胞母细胞，以诱导PD-1的表达，结果表明外泌体PD-L1以剂量依赖的方式显著抑制CD3/CD28诱导的T细胞ERK磷酸化和核因子κB（nuclear factor kappa B, NF-κB）的激活以及PHA诱导的IL-2的分泌。其他癌细胞系研究如结肠癌RKO和肺癌HCC827的exoPD-L1也可以参与免疫检查点途径来阻断T细胞的激活。这些数据支持exo-PD-L1可抑制T细胞激活，为癌症中一种重要的新的免疫抑制机制^[31]^。

PD-L1系统性地抑制抗肿瘤免疫反应，其基因阻断促进引流淋巴结中的T细胞活性，从而诱导系统性抗肿瘤免疫和记忆^[32]^。遗传学上两个重要的外泌体生物发生基因：*Rab27a*和*nSMNase2*，在外泌体生物发生和PD-L1分泌中起着关键作用^[32]^。从基因上阻止外泌体的生物发生或敲除*PD-L1*基因可以强烈促进T细胞的激活、增殖和杀伤能力来逆转表型，而随着外源exoPD-L1的引入，这种效应再次被逆转^[32]^。这两种基因各自表达的蛋白产物都代表了潜在的治疗靶点，然而针对Rab27a和nSMNase2的小分子抑制剂虽然已经存在，但无法抑制癌细胞系中的外泌体释放，还需要更多的研究针对这些目标探索更有效的小分子。

## 4 肿瘤细胞exoPD-L1对NSCLC免疫治疗的作用

### 4.1 exoPD-L1作为潜在的生物标志物

外泌体积极参与癌症的发展、转移和耐药，这使它们成为癌症筛查、诊断和监测的生物标志物^[33]^。近几年，外泌体蛋白在诊断许多癌症方面表现出非常高的敏感性和特异性。而exoPD-L1蛋白被人们研究较多，有研究^[33]^发现阻断PD-1/PD-L1通路可以逆转肿瘤微环境，诱导内源性抗肿瘤免疫反应。事实上，PD-1/PD-L1免疫疗法已经在临床试验中显示了对NSCLC患者的益处。

Liu等^[34]^开发了一种表面等离子体共振（surface plasmon resonance, SPR）生物传感器，用于捕获外泌体并表征用于癌症诊断的外泌体蛋白。在收集的肺癌患者的血清样本中观察到exoPD-L1水平高于正常对照组，这表明exoPD-L1可能是肺癌诊疗的有效生物标志物。但应该指出的是，exoPD-L1作为诊断生物标志物的效用需要在大型患者队列、具有其他肺癌组织学特征的患者（如鳞状NSCLC、小细胞肺癌）以及疾病早期阶段（如Ⅰ期）的患者中进一步验证。

Yang等^[35]^收集了51例晚期NSCLC患者的配对组织标本和血液标本，检测晚期NSCLC患者ICIs治疗2个月后血液中PD-L1表达的动态变化，包括PD-L1 mRNA、exoPD-L1蛋白和sPD-L1的变化。在40例晚期NSCLC患者中，PD-L1 mRNA变化倍数≥2.04的患者具有更好的PFS、OS和最佳总体疗效（best of response, BOR）。此外，在一组21例晚期NSCLC患者中，发现exoPD-L1≥1.86的倍数变化与更好的疗效和OS相关，而sPD-L1的动态变化与疗效和OS无关。这表明ICIs治疗早期PD-L1 mRNA和/或exoPD-L1表达升高可作为晚期NSCLC患者疗效和OS的阳性生物标志物。此外，PD-L1 mRNA和exoPD-L1的组合可以筛选更好的患者，以获得ICIs治疗的潜在益处。

进一步的研究^[35, 36]^表明，PD-L1阳性外泌体中含有的miR-21有可能成为区分NSCLC患者和健康对照人员之间差异的生物标志物。Yang等^[37]^用免疫芯片检测了人血清中含EGFR或PD-L1外泌体的miR-21和甲状腺转录因子-1（thyriod transcription factor-1, TTF-1）mRNA的表达，获得了区分正常对照人员和NSCLC患者的绝对敏感性和特异性。在A549 *EGFR*阳性外泌体中，miR-21和TTF-1的mRNA水平分别是BEAS-2B细胞的1.6倍和2.8倍。同时，来自A549细胞的PD-L1阳性外泌体的miR-21和TTF-1 mRNA水平分别是BEAS-2B细胞的5.3倍和5.9倍。这些结果表明，PD-L1阳性外泌体miR-21和TTF-1 mRNA具有比*EGFR*阳性外泌体更好的肿瘤识别性能。这提示*EGFR*阳性和PD-L1阳性外泌体miR-21和TTF-1 mRNA是区分NSCLC患者和健康对照人员的有效血清生物标志物。在胞外体携带的各种类型的标志物中，RNA特别是microRNAs已被证明是在癌症筛查、诊断和预后方面有前景的新生物标志物^[38]^。

### 4.2 exoPD-L1阻断有效抑制肿瘤进展

众所周知，PD-1/PD-L1阻断可以激活T细胞，但对于exoPD-L1在抗PD-1/PD-L1治疗有效率相对较低中的作用知之甚少。新的证据表明，TEX有助于肿瘤的发生，并调节肿瘤的生长、血管生成、免疫调节、转移和耐药。因此它们是新的和有效的癌症液体活检的生物标志物。exoPD-L1在许多肿瘤包括胶质母细胞瘤、头颈癌、乳腺癌、肺癌等中表达^[39-42]^。有研究^[40]^发现癌细胞来源的外泌体表达PD-L1，并且这些外泌体可以支持肺癌的肿瘤进展，并且循环exoPD-L1在抗PD-1/PD-L1阻断治疗中发挥重要作用。

Ricklefs等^[39]^筛选85例初诊为NSCLC的患者和27名健康体检者为研究对象，分析exoPD-L1、sPD-L1、PD-L1免疫组化特征与临床病理特征的相关性。结果显示，NSCLC患者，特别是晚期患者，相比健康对照组，exoPD-L1水平更高。此外，在NSCLC患者中，exoPD-L1含量高与肿瘤大小、阳性淋巴结状态、远处转移和肿瘤原发灶-淋巴结-转移（tumor-node-metastasis, TNM）分期有关。而NSCLC患者血清sPD-L1水平与健康体检者无明显差异，与肿瘤大小（> 2.5 cm）外的其他临床病理特征均无相关性（*P* > 0.05）。总之，exoPD-L1与NSCLC疾病进展相关，包括肿瘤大小、淋巴结状态、转移和TNM分期。

Kim等^[40]^检测了PD-L1阳性外泌体处理后CD8^+^ T细胞的增殖和凋亡情况。CD8^+^ T细胞在CD3/CD28刺激下增殖，但外泌体处理不影响其增殖，然而，外泌体治疗确实以剂量依赖的方式减少了CD8^+^ T细胞的总数。与这些结果一致的是，用外泌体处理细胞显著诱导了CD8^+^ T细胞的凋亡，当用PD-L1阴性外泌体处理细胞时，CD8^+^ T细胞的凋亡率降低，表明PD-L1阳性外泌体通过PD-1/PD-L1的相互作用促进CD8^+^ T细胞的凋亡^[41]^。此外，从NSCLC患者血液中分离的外泌体上存在PD-L1，PD-L1阳性的外泌体通过抑制细胞因子和诱导CD8^+^ T细胞的凋亡在肿瘤免疫逃逸中发挥重要作用^[42]^。

Del Re等^[43]^研究首次证明了在抗PD-1抗体治疗过程中exoPD-L1表达的变化，对患有局部晚期或转移性黑色素瘤或NSCLC的患者给予尼伏鲁单抗或培溴利珠单抗治疗2个月后检测PD-L1外泌体mRNA水平。结果显示，在治疗有效的患者中，血浆来源的外泌体中的PD-L1水平显著降低，而在疾病进展的患者中，PD-L1水平上升，而在疾病稳定患者中没有观察到明显的变化。这项研究表明，exoPD-L1与治疗反应显著相关。

用抗PD-1受体阻断抗体预处理胶质母细胞瘤EV几乎逆转了T细胞的抑制并阻止了肿瘤的进展，而抗PD-1抗体可逆转这些作用并阻止肿瘤进展。抗PD-1抗体可逆转外泌体介导的胶质母细胞瘤中T细胞活化的阻断，表明这些抗PD-1抗体与exoPD-L1竞争结合PD-1 T细胞^[39]^。有研究^[19]^还发现转移性黑色素瘤来源的外泌体上的PD-L1抑制CD8^+^ T细胞的激活，促进肿瘤生长，这些作用可以被抗PD-1抗体治疗阻断。令人惊讶的是，尽管细胞表面的PD-L1很低或不存在，但exoPD-L1可能是由其亲代肿瘤细胞高度分泌的，这些细胞对抗PD-L1治疗具有耐药性^[32]^。总之，exoPD-L1在免疫抑制和耐药方面起着关键作用。在NSCLC患者和黑色素瘤患者中，也发现胞外PD-L1 mRNA水平与抗PD-1 ICIs Nivolumab和Pembrolizumab的反应相关^[38]^。这些发现表明，exoPD-L1代表着一个尚未探索的治疗靶点，消除外泌体可能是抗PD-1/PD-L1抗体增强的可行且必要的伴随疗法^[44, 45]^。

## 5 小结

自2014年Pembrolizumab首次获批用于治疗晚期或不可切除的黑色素瘤以来，已有七种靶向PD-1或PD-L1的抗体获FDA批准用于治疗实体瘤或血液肿瘤疾病（淋巴瘤）。这些ICIs大大提高了许多晚期恶性肿瘤患者的生存率，但并非全部。从一种癌症到另一种癌症的临床反应差异很大，反应的持久性也因患者而异。展望未来，在抗PD-1/PD-L1免疫检查点治疗过程中，不仅要考虑细胞表面PD-L1，还要考虑exoPD-L1^[46]^。阻断外泌体释放或抑制肿瘤微环境中外泌体介导的细胞串扰可能抑制肿瘤微环境有利因素的发展，这不失为一种新的肺癌免疫治疗方法。总之，exoPD-L1作为生物标志物提供了理论依据，但需要进一步扩展，使外泌体的液体活检成为可能。未来更需要专门针对exoPD-L1蛋白开展研究，探索ICIs和exoPD-L1抑制剂的联合作用，以开发针对肺癌细胞免疫逃逸的新策略，并加强肺癌免疫治疗的效果，为NSCLC患者免疫治疗突破瓶颈探索新的机遇。
